# Gastrointestinal implications in COVID-19

**DOI:** 10.1186/s12879-021-06824-y

**Published:** 2021-11-04

**Authors:** Ghazal Zoghi, Seyed Hamid Moosavy, Sadegh Yavarian, Mehdi HasaniAzad, Farid Khorrami, Masoud Sharegi Brojeni, Masoumeh Kheirandish

**Affiliations:** 1 Endocrinology and Metabolism Research Center, Hormozgan University of Medical Sciences, Bandar Abbas, Iran; 2 Student Research Committee, Faculty of Medicine, Hormozgan University of Medical Sciences, Bandar Abbas, Iran; 3 Infectious and Tropical Diseases Research Center, Hormozgan Health Institute, Hormozgan University of Medical Sciences, Bandar Abbas, Iran; 4 Department of Health Information Technology, Faculty of Para-Medicine, Hormozgan University of Medical Sciences, Bandar Abbas, Iran

**Keywords:** Coronavirus, SARS-CoV-2, Gastrointestinal symptoms

## Abstract

**Background:**

Coronavirus disease 2019 (COVID-19 patients mostly present with respiratory symptoms; however, gastrointestinal (GI) manifestations can also be seen either alone or along with respiratory symptoms. We aimed to evaluate the GI symptoms related to COVID-19.

**Methods:**

This cross-sectional study retrospectively evaluated the medical files of 507 patients with confirmed or highly probable COVID-19. Based on their symptoms, patients were categorized into four groups: with GI symptoms alone (GIA), with respiratory symptoms alone (RA), with both GI and respiratory symptoms (GIR), and without GI or respiratory symptoms (WGIR).

**Results:**

Of the 507 COVID-19 patients, 47.9% had at least one GI symptom; the most common was nausea and/or vomiting (31.6%). Patients in the GIA group were significantly older than those in the RA (P = 0.041) and GRI (P = 0.004) groups (54.70 ± 18.14 vs. 48.68 ± 14.67 and 46.80 ± 17.17 years, respectively). Groups were homogeneous with respect to gender. Leukopenia and lymphopenia were both less frequent in patients with GI symptoms compared to those without GI symptoms. Positive RT-PCR was significantly less frequent among patients with GI symptoms (44% vs. 100%, P < 0.001). Although mortality was lower in patients with GI symptoms (9.1%) in comparison with those without GI symptoms (13.3%), the difference was not statistically significant (P = 0.134).

**Conclusion:**

The typical respiratory symptoms of COVID-19 are quite commonly accompanied by GI symptoms, with nausea and/or vomiting being the most prevalent. A subgroup of COVID-19 patients may exclusively present with GI symptoms. Special attention should be paid to these patients in order to avoid misdiagnosis or delayed treatment.

## Background

Coronavirus disease 2019 (COVID-19) which has grown to pandemic proportions is caused by severe acute respiratory syndrome coronavirus 2 (SARS-CoV-2) [1]. Typical manifestations of COVID-19 include fever and respiratory symptoms; however, extrapulmonary manifestations, including acute coronary syndromes, neurologic illnesses, dermatologic complications, thrombotic complications, acute kidney injury, and endocrine disorders have also been reported [2]. Some COVID-19 patients may present with gastrointestinal (GI) symptoms such as abdominal pain, nausea and/or vomiting, and diarrhea along with the usual respiratory symptoms [3]. The prevalence of diarrhea and nausea or vomiting in COVID-19 patients has been reported as 3.8% and 5%, respectively in a multicenter study conducted in China [4]. Moreover, in another study, nausea and diarrhea were present at the onset of COVID-19 in approximately 10% of the patients [5]. Additionally, ageusia has been reported in 15.7% of patients with COVID-19 in a study on 3191 patients in Korea [6].

Angiotensin converting enzyme 2 receptor (ACE2) is thought to be responsible for the entrance of SARS-CoV-2 into the host cells. This viral receptor has been found to be abundantly expressed in GI epithelial cells [7, 8]. Moreover, SARS-CoV-2 RNA has been identified in fecal specimens and rectal swabs of the infected patients long after its clearance from the respiratory system, confirming the presence of the virus in the GI tract and suggesting a potential fecal–oral route of transmission [9–12]. Also, the potential transmission of COVID-19 through endoscopy by close contact with oral and colonic contents has been described [13, 14].

Early diagnosis and timely management of COVID-19 patients play a critical role in their outcomes. On the other hand, isolation of the infected individuals is an important strategy to prevent the spread of the disease. Therefore, characterization of the wide spectrum of clinical manifestations of COVID-19 can contribute to its identification. Furthermore, estimation of the prevalence of GI symptoms in COVID-19 patients will draw the attention of clinicians and GI practitioners and enhance their vigilance towards patients with these symptoms leading to the reduction of COVID-19 underdiagnosis and the subsequent transmission of the disease in the community. In the current study, we aimed to evaluate the involvement of the GI system in COVID-19 patients with the hope that the results of this study would be helpful for disease control.

## Methods

### Participants

In this cross-sectional study, we retrospectively evaluated patients aged ≥ 18 years with confirmed (by reverse transcriptase polymerase chain reaction [RT-PCR]) or highly probable (based on clinical and computer tomography [CT] findings) COVID-19 admitted to Shahid Mohammadi Hospital, Bandar Abbas, Iran from February 20 to August 20, 2020. Pregnant women and patients with incomplete medical files were excluded.

### Study design

Medical charts of 507 patients with confirmed or highly probable COVID-19 were reviewed. Data were extracted from patients’ medical files including age, gender, and the following variables:

- Risk factors (recent travel, animal contact, smoking, opium use, and alcohol consumption);
- General signs and symptoms (fever, shivering, headache, dizziness, perspiration, anosmia/ageusia, muscle pain, joint pain, fatigue, seizure, lymphadenopathy, altered consciousness, conjunctivitis, skin rash/ulcer, palpitation, and hemorrhage);
- Respiratory signs and symptoms (cough, shortness of breath, intercostal retraction, sore throat, rhinorrhea, chest pain, sputum production, and bloody sputum/hemoptysis);
- GI symptoms (abdominal pain, nausea and/or vomiting, diarrhea, anorexia, GI bleeding, and constipation);
- Underlying illnesses (chronic cardiovascular disease, hypertension, hyperthyroidism, hypothyroidism, glucose-6-phosphate dehydrogenase [G6PD] deficiency, hyperlipidemia, malignant neoplasm, chronic hematologic disease, chronic neurologic disorder, obesity, chronic pulmonary disease, diabetes, asthma, chronic kidney disease, rheumatologic disorder, liver disease, dementia, and malnutrition);
- Vital signs on admission (body temperature, heart rate [HR], respiratory rate [RR], systolic blood pressure [SBP], and diastolic blood pressure [DBP]);
- Laboratory test results (RT-PCR, hemoglobin [Hb], white blood cell [WBC] count and differentials [percentage of lymphocytes and neutrophils], hematocrit [Hct], platelet count, erythrocyte sedimentation rate [ESR], partial thromboplastin time [PTT], prothrombin time [PT], international normalized ratio [INR], and serum alanine aminotransferase [ALT], aspartate aminotransferase [AST], total bilirubin, plasma glucose [PG], blood urea nitrogen [BUN], creatinine [Cr], lactate dehydrogenase [LDH], C-reactive protein [CRP], troponin, sodium [Na], potassium [K], and magnesium [Mg]); and
- Computed tomography (CT) scan findings (local patchy shadowing, interstitial abnormalities, peripherally distributed opacities, linear opacities, discrete nodule, unilateral/bilateral involvement, pleural effusion, cavitation, pneumothorax, and lymphadenopathy).

Intensive care unit (ICU) admission, assisted ventilation, oxygen therapy, and outcomes (death or survival) were also recorded. Those symptoms of patients that were present upon their admission or during hospital stay have been taken into account.

Of note, RT-PCR tests had been performed on specimens from nasopharyngeal swabs using the STANDARD M nCoV Real-Time Detection Kit (manufactured by SD Biosensor Inc., South Korea). The kit is based on TaqMan probe technology targeting RdRp and E genes. According to the manufacturer’s instructions, the cycle threshold (Ct) for RdRp and E genes were ≤ 36.

Based on their symptoms, patients were categorized into four groups: with GI symptoms alone (GIA), including abdominal pain, nausea/vomiting, diarrhea, GI bleeding, constipation, or anorexia without any respiratory symptoms, patients with respiratory symptoms alone (RA), with both GI and respiratory symptoms (GIR), and without GI or respiratory symptoms (WGIR).

### Data analysis

The Statistical Package for the Social Sciences (SPSS) software (version 25.0, Armonk, NY: IBM Corp., US) was used for data analysis. Mean, standard deviation, frequency, and percentages were used to describe the results. Chi-squared test and Fisher’s exact test were used to compare qualitative variables among groups. To compare the means of quantitative variables, first the test for homogeneity of variances was performed. In case of homogeneous variances, one-way analysis of variance (ANOVA) was used to compare means; otherwise, robust tests of equality of means were used. Accordingly, pairwise comparisons were done using the appropriate post hoc tests. P-values < 0.05 were regarded as statistically significant.

## Results

Of the 507 patients included in the current study, 289 (57%) were male and 218 (43%) were female. Their mean age was 49.58 ± 16.49 (18–91) years. Regarding the four defined categories, 92 patients (18.1%) were in the GIA, 184 (36.3%) in the RA, 151 (29.8%) in the GIR, and 80 (15.8%) in the WGIR groups. Age was significantly associated with patients’ symptoms (P = 0.002); patients in the GIA group were significantly older than those in the RA (P = 0.041) and GRI (P = 0.004) groups (54.70 ± 18.14 vs. 48.68 ± 14.67 and 46.80 ± 17.17 years, respectively). The mean age of the WGRI patients was 50.99 ± 15.95 years. However, taking GI symptoms into account (regardless of the presence of respiratory symptoms), there was no significant age difference between patients with and without GI symptoms (P = 0.783). With respect to gender, 52 (56.6%) in the GIA group, 110 (59.8%) in the RA group, 87 (57.6%) in the GIR group, and 40 (50%) in the WGIR group were male (P = 0.529). Moreover, disregarding the respiratory symptoms, 139 (57.2%) of the patients with GI symptoms and 150 (56.8%) of those without GI symptoms were male (P = 0.931).

Table 1 shows the comparison of primary vital signs and laboratory test results among the four groups of patients. HR, DBP, WBC, percentages of lymphocytes and neutrophils, BUN, and ESR differed significantly among groups. In general, 83/479 patients (17.3%) and 5/79 (6.3%) in the GIA group, 38/182 (20.9%) in the RA group, 25/138 (18.1%) in the GIR group, and 15/80 (18.8%) in the WGIR group had leukopenia. A statistically significant difference was found among groups in this respect (P = 0.038). In addition, generally 185/479 patients (38.6%) and 25/79 (31.6%) in the GIA group, 78/182 (57.1%) in the RA group, 51/138 (37%) in the GIR group, and 31/80 (38.8%) in the WGIR group had lymphopenia (P = 0.367). When patients with and without GI symptoms (regardless of the presence of respiratory symptoms) were compared in terms of leukopenia and lymphopenia, both were higher in patients without GI symptoms (20.2% vs. 13.8% and 41.6% vs. 35%, respectively); nonetheless, the differences were not statistically significant (P = 0.065 and P = 0.141, respectively).

**Table 1:** Comparison of laboratory test results and primary vital signs in COVID-19 patients with GI or respiratory symptoms

Variable	Total	GIA	RA	GIR	WGIR	P-value*
	Mean ± SD	Mean ± SD	Mean ± SD	Mean ± SD	Mean ± SD	
Temperature (°C)	37.22 ± 0.72	37.17 ± 0.83	37.24 ± 0.68	37.21 ± 0.71	37.22 ± 0.67	0.886
HR (bpm)	83.71 ± 17.94	89.71 ± 20.56^a,b^	81.82 ± 17.49^a,c^	86.56 ± 16.85^d^	75.22 ± 13.62^b,c,d^	< 0.001^†^
RR (/min)	20.07 ± 6.96	19.92 ± 6.36	19.13 ± 2.45	21.23 ± 10.04	20.19 ± 6.96	0.065^†^
SBP (mmHg)	119.46 ± 19.11	117.94 ± 20.95	120.02 ± 17.45	117.51 ± 19.26	123.59 ± 19.88	0.109
DBP (mmHg)	74.73 ± 12.35	75.07 ± 14.06	74.61 ± 10.62	72.81 ± 11.35^a^	78.21 ± 14.95^a^	0.017
Hb (g/dL)	12.33 ± 2.12	11.90 ± 2.47	12.47 ± 1.98	12.50 ± 2.17	12.15 ± 1.90	0.187^†^
WBC (/µL)	7282.25 ± 6026.07	9445.57 ± 9457.23^a^	6306.59 ± 3548.21^a^	7234.06 ± 4308.89	7448.75 ± 7966.51	0.011^†^
Lymphocyte (%)	23.68 ± 12.79	19.70 ± 13.82^a^	25.13 ± 12.26^a^	23.95 ± 12.63	23.88 ± 12.59	0.019
Neutrophil (%)	68.44 ± 14.95	73.64 ± 15.50^a,b^	66.57 ± 14.57^a^	67.39 ± 14.56^b^	69.29 ± 14.94	0.005
Hct (%)	37.25 ± 5.73	35.68 ± 7.49	37.66 ± 5.06	37.63 ± 5.84	37.23 ± 4.74	0.182^†^
Platelet (/µL)	214,811.84 ± 93,586.39	224,787.50 ± 86,840.71	217,412.43 ± 90,167.11	206,345.59 ± 96,070.53	213,475.00 ± 103,255.24	0.538
PTT (s)	35.92 ± 17.95	34.50 ± 11.85	34.73 ± 9.71	38.19 ± 26.24	34.01 ± 5.52	0.535
PT (s)	15.19 ± 12.82	15.52 ± 8.49	13.76 ± 3.04	17.55 ± 22.61	14.15 ± 4.60	0.204^†^
INR	1.20 ± 0.95	1.39 ± 1.05	1.19 ± 1.26	1.11 ± 0.29	1.19 ± 0.72	0.377
ALT (U/L)	45.10 ± 36.42	55.03 ± 60.52	40.52 ± 22.97	45.43 ± 30.35	45.15 ± 39.52	0.183^†^
AST (U/L)	51.64 ± 61.79	72.18 ± 1118.98	46.37 ± 37.86	46.71 ± 37.47	52.49 ± 56.60	0.355^†^
Total bilirubin (mg/dL)	1.41 ± 2.92	1.95 ± 4.12	0.98 ± 1.04	1.67 ± 4.03	1.30 ± 1.67	0.262^†^
PG (mg/dL)	132.25 ± 83.99	127.16 ± 72.20	137.94 ± 85.33	121.44 ± 71.45	142.50 ± 107.29	0.362^†^
BUN (mg/dL)	33.57 ± 34.14	49.85 ± 60.75^a,b^	29.45 ± 19.82^a^	32.72 ± 23.98	27.31 ± 31.17^b^	0.019^†^
Cr (mg/dL)	1.31 ± 1.71	1.61 ± 2.27	1.13 ± 0.87	1.34 ± 2.02	1.32 ± 1.81	0.211^†^
LDH (mg/dL)	540.26 ± 813.20	592.14 ± 864.66	476.16 ± 275.08	690.62 ± 1323.84	395.86 ± 252.51	0.088
ESR (mm/h)	37.97 ± 27.08	31.88 ± 25.10	42.09 ± 27.99^a^	33.39 ± 26.15^a^	40.18 ± 26.50	0.017
CRP (mg/L)	7.31 ± 14.29	16.26 ± 23.96	6.18 ± 12.09	15.74 ± 15.36	4.35 ± 12.70	0.068^†^
Troponin (µg/dL)	330.6 9 ± 3426.62	1155.98 ± 7288.37	53.73 ± 383.07	340.67 ± 2561.97	41.24 ± 168.17	0.588^†^
Na (mEq/L)	138.06 ± 5.04	137.82 ± 4.76	138.48 ± 5.56	137.98 ± 4.34	137.49 ± 5.26	0.525
K (mEq/L)	4.22 ± 0.56	4.23 ± 0.71	4.28 ± 0.56	4.15 ± 0.49	4.18 ± 0.52	0.272
Mg (mEq/L)	2.03 ± 0.61	1.82 ± 0.28	2.00 ± 0.39	1.77 ± 0.35	2.11 ± 0.79	0.585

*RA* respiratory alone, *GIA* gastrointestinal alone, *GIR* gastrointestinal and respiratory, *WGIR* without gastrointestinal or respiratory, *SD* standard deviation, *HR* heart rate, *RR* respiratory rate, *SBP* systolic blood pressure, *DBP* diastolic blood pressure, *Hb* hemoglobin, *WBC* white blood cells, *Hct* hematocrit, *PTT* partial thromboplastin time, *PT* prothrombin time, *INR* international normalized ratio, *ALT* alanine aminotransferase, *AST* aspartate aminotransferase, *PG* plasma glucose, *BUN* blood urea nitrogen, *Cr* creatinine, *LDH* lactate dehydrogenase, *ESR* erythrocyte sedimentation rate, *CRP* C-reactive protein, *Na* sodium, *K* potassium, *Mg* magnesium

*Analyzed by one-way ANOVA

^†^Analyzed by robust tests of equality of means

^a–d^Statistically significant differences by post-hoc analysis

Comparison of the underlying diseases showed that there was a significant correlation between the symptom category of COVID-19 patients and the presence of G6PD deficiency (P = 0.025), malignant neoplasm (P = 0.003), rheumatologic disorder (P = 0.015), and liver disease (P = 0.005) (Table 2). None of the patients had dementia or malnutrition.

**Table 2:** Comparison of underlying diseases in COVID-19 patients with GI or respiratory symptoms

Variable	Total	GIA (n = 92)	RA (n = 184)	GIR (n = 151)	WGIR (n = 80)	P-value*
	N (%)	N (%)	N (%)	N (%)	N (%)	
Chronic cardiovascular disease	60 (11.8)	16 (17.4)	23 (12.5)	11 (7.3)	10 (12.5)	0.120
Hypertension	128 (25.3)	28 (30.4)	43 (23.5)	35 (23.2)	22 (27.5)	0.539
Hyperthyroidism	1 (0.2)	0 (0)	1 (0.5)	0 (0)	0 (0)	1.000^†^
Hypothyroidism	13 (2.6)	2 (2.2)	5 (2.7)	5 (3.3)	1 (1.3)	0.868^†^
G6PD deficiency	5 (1.0)	0 (0)	2 (1.1)	0 (0)	3 (3.8)	0.025^†^
Hyperlipidemia	30 (5.9)	6 (6.5)	13 (7.1)	9 (6.0)	2 (2.5)	0.537
Malignant neoplasm	8 (1.6)	5 (5.4)	0 (0)	3 (2.0)	0 (0)	0.003^†^
Chronic hematologic disease	9 (1.8)	2 (2.2)	3 (1.6)	2 (1.3)	2 (2.5)	0.845^†^
Chronic neurologic disorder	17 (3.4)	4 (4.3)	5 (2.7)	5 (3.3)	3 (3.8)	0.869^†^
Obesity	0 (0)	3 (1.6)	0 (0)	0 (0)	0 (0)	0.268^†^
Chronic pulmonary disease	12 (2.4)	4 (4.3)	4 (2.2)	3 (2.0)	1 (1.3)	0.608^†^
Diabetes	116 (22.9)	24 (26.1)	46 (25.0)	24 (15.9)	22 (27.5)	0.090
Asthma	27 (5.3)	3 (3.3)	15 (8.2)	6 (4.0)	3 (3.8)	0.264^†^
Chronic kidney disease	29 (5.7)	9 (9.8)	7 (3.8)	6 (4.0)	7 (8.8)	0.099
Rheumatologic disorder	7 (1.4)	1 (1.1)	0 (0)	2 (1.3)	4 (5.0)	0.015^†^
Liver disease	10 (2.0)	6 (6.5)	1 (0.5)	1 (0.7)	2 (2.5)	0.005^†^

*RA* respiratory alone, *GIA* gastrointestinal alone, *GIR* gastrointestinal and respiratory, *WGIR* without gastrointestinal or respiratory, *N* number, *G6PD* glucose-6-phosphate dehydrogenase

*Analyzed by Chi-squared test

^†^Analyzed by Fisher’s exact test

As for risk factors and general signs and symptoms, fever, shivering, headache, anosmia/ageusia, fatigue, recent travel, animal contact, smoking, and opium use were significantly correlated with patients’ category of symptoms (Table 3). Conjunctivitis, skin rash/ulcer, palpitation, and hemorrhage were not present in any of the patients. Moreover, RT-PCR results, noninvasive ventilation, ICU admission, oxygen therapy, and outcome (death or survival) were significantly associated with patients’ symptoms (Table 3). These variables were also compared between patients with and without GI symptoms, disregarding the presence of respiratory symptoms (results not shown in the table). Positive RT-PCR was significantly less frequent among patients with GI symptoms compared to those without these symptoms (P < 0.001). Noninvasive ventilation was performed less frequently on patients with GI symptoms (P = 0.016). Oxygen therapy was significantly higher in patients with GI symptoms and ICU admission in those without GI symptoms (P < 0.001 and P = 0.005, respectively). Finally, although mortality was lower in patients with GI symptoms (9.1%) in comparison with those without GI symptoms (13.3%), the difference was not statistically significant (P = 0.134).

**Table 3:** Comparison of risk factors, general signs and symptoms, RT-PCR results, ICU admission, assisted ventilation, oxygen therapy, and outcomes in COVID-19 patients with GI or respiratory symptoms

Variable	Total	GIA (n = 92)	RA (n = 184)	GIR (n = 151)	WGIR (n = 80)	P-value*
	N (%)	N (%)	N (%)	N (%)	N (%)	
General signs and symptoms						
Fever	249 (49.1)	32 (34.8)	97 (52.7)	90 (59.6)	30 (37.5)	< 0.001
Shivering	78 (15.4)	4 (4.3)	28 (15.2)	30 (19.9)	16 (20.0)	0.007
Headache	46 (9.1)	11 (12.0)	13 (7.1)	21 (13.9)	1 (1.3)	0.007
Dizziness	20 (3.9)	5 (5.4)	4 (2.2)	10 (6.6)	1 (1.3)	0.097^†^
Perspiration	6 (1.2)	1 (1.1)	2 (1.1)	2 (1.3)	1 (1.3)	1.000^†^
Anosmia/ageusia	36 (7.1)	2 (2.2)	15 (8.2)	16 (10.6)	3 (3.8)	0.049
Muscle pain	126 (24.9)	19 (20.7)	46 (25.0)	39 (25.8)	22 (27.5)	0.741
Joint pain	28 (5.5)	3 (3.3)	13 (7.1)	11 (7.3)	1 (1.3)	0.142
Fatigue	44 (8.7)	15 (16.3)	0 (0)	29 (19.2)	0 (0)	< 0.001
Seizure	2 (0.4)	0 (0)	0 (0)	1 (0.7)	1 (1.3)	0.297^†^
Lymphadenopathy	1 (0.2)	0 (0)	1 (0.5)	0 (0)	0 (0)	1.000^†^
Altered consciousness	2 (0.4)	1 (1.1)	0 (0)	0 (0)	1 (1.3)	0.115^†^
Risk factors						
Recent travel	9 (1.8)	1 (1.1)	3 (1.6)	2 (1.3)	3 (3.8)	< 0.001^†^
Animal contact	3 (0.6)	0 (0)	3 (1.6)	0 (0)	0 (0)	< 0.001^†^
Smoking	38 (7.5)	10 (10.9)	12 (6.50	16 (10.6)	0 (0)	0.016
Opium use	15 (3.0)	5 (5.4)	2 (1.1)	8 (5.3)	0 (0)	0.015^†^
Alcohol consumption	3 (0.6)	1 (1.1)	0 (0)	2 (1.3)	0 (0)	0.316^†^
RT-PCR results						
Positive	371 (73.2)	27 (29.3)	184 (100)	80 (53.0)	80 (100)	< 0.001
Negative	136 (26.8)	65 (70.7)	0 (0)	71 (47.0)	0 (0)	
Assisted ventilation						
Noninvasive ventilation	30 (5.9)	4 (4.3)	10 (5.4)	4 (2.6)	12 (15.0)	0.002
Invasive ventilation	45 (8.9)	9 (9.8)	16 (8.7)	8 (5.3)	12 (15.0)	0.102
Measures during hospitalization						
ICU admission	61 (12.0)	12 (13.0)	22 (12.0)	7 (4.6)	20 (25.0)	< 0.001
Oxygen therapy	308 (60.7)	67 (72.8)	105 (57.1)	102 (67.5)	34 (42.5)	< 0.001
Outcome						
Death	57 (11.2)	13 (14.1)	20 (10.9)	9 (6.0)	15 (18.8)	0.023
Survival	450 (88.8)	79 (85.9)	164 (89.1)	142 (94.0)	65 (81.3)	

*RA* respiratory alone, *GIA* gastrointestinal alone, *GIR* gastrointestinal and respiratory, *WGIR* without gastrointestinal or respiratory, *N* number, *RT-PCR* reverse transcriptase polymerase chain reaction, *ICU* intensive care unit

*Analyzed by Chi-squared test

^†^Analyzed by Fisher’s exact test

In general, 243 patients (47.9%) had at least one GI symptom irrespective of the presence of respiratory symptoms and 335 (66.1%) had at least one respiratory sign or symptom regardless of the presence of GI symptoms. Besides, 190 patients (37.5%) had only one GI symptom (abdominal pain, nausea and/or vomiting, and diarrhea), 16 (3.2%) had abdominal pain and nausea and/or vomiting, 5 (1%) had abdominal pain and diarrhea, 28 (5.5%) had nausea and/or vomiting and diarrhea, and 4 (0.8%) had all the three GI symptoms. Anorexia, GI bleeding, and constipation were not present in any of the patients. By comparing respiratory signs and symptoms between patients in the RA and GIR groups, cough (P = 0.024), shortness of breath (P < 0.001), rhinorrhea (P = 0.029), and sputum production (P = 0.027) were significantly more frequent in patients of the GIR group, while intercostal retraction was significantly more common in the RA group (P < 0.001) (Table 4). The GI symptoms and CT scan findings were compared between the GIA and GIR groups. Among the GI symptoms, only the difference in the frequency of abdominal pain was statistically significant; it was significantly higher in the GIA group compared to the GIR group (P = 0.008). Notably, pleural effusion, cavitation, pneumothorax, and lymphadenopathy were not observed in the CT scans of any of the participants.

**Table 4:** Comparison of CT scan findings and respiratory signs and symptoms, and GI symptoms in COVID-19 patients of different symptom categories

Variable	GIA (n = 92)	RA (n = 184)	GIR (n = 151)	P-value*
	N (%)	N (%)	N (%)	
Respiratory signs and symptoms				
Cough		177 (96.2)	136 (90.1)	0.024
Shortness of breath		11 (6.0)	81 (53.6)	< 0.001
Intercostal retraction		46 (25.0)	0 (0)	< 0.001
Sore throat		16 (8.7)	12 (7.9)	0.805
Rhinorrhea		1 (0.5)	6 (4.0)	0.029^†^
Chest pain		11 (6.0)	16 (10.6)	0.122
Sputum production		19 (10.3)	26 (17.2)	0.027
Bloody sputum/hemoptysis		3 (1.6)	4 (2.6)	0.493^†^
GI symptoms				
Abdominal pain	27 (29.3)		23 (15.2)	0.008
Nausea and/or vomiting	64 (69.6)		96 (63.6)	0.340
Diarrhea	33 (35.9)		57 (37.7)	0.769
CT scan findings				
Local patchy shadowing	1 (1.3)		2 (1.4)	1.000^†^
Interstitial abnormalities	2 (2.5)		1 (0.7)	0.296^†^
Peripheral opacities	1 (1.3)		6 (4.3)	0.426^†^
Linear opacities	2 (2.5)		2 (1.4)	0.621^†^
Discrete nodule	1 (1.3)		4 (2.9)	0.656^†^
Unilateral involvement	0 (0)		3 (2.1)	0.555^†^
Bilateral involvement	3 (3.8)		13 (9.3)	0.134

*RA* respiratory alone, *GIA* gastrointestinal alone, *GIR* gastrointestinal and respiratory, *WGIR* without gastrointestinal or respiratory, *N* number, *CT* computed tomography, *GI* gastrointestinal

*Analyzed by Chi-squared test

^†^Analyzed by Fisher’s exact test

## Discussion

The results of this study showed that approximately half of the patients had at least one GI symptom. The most common GI symptom in COVID-19 patients was nausea and/or vomiting (31.6%) followed by diarrhea (17.8%) and abdominal pain (9.9%). GI symptoms have also been reported in previous studies as part of COVID-19 presentations with high variability of prevalence, though. Likewise, Pan et al. reported that nearly half of the patients with COVID-19 had digestive symptoms; nonetheless, anorexia and diarrhea were the most common symptoms in their study [15]. Additionally, GI symptoms were found in 39.6% of the COVID-19 patients in a cohort study, including nausea (17.3%), diarrhea (12.9%), and vomiting (5%) [16]. A large study from China, consisting of 1099 patients, reported diarrhea in 3.8% and nausea or vomiting in 5% [15]. The frequency of diarrhea and nausea and/or vomiting ranged from 2 to 10.1% and 1 to 10.1%, respectively in other studies [16–22]. However, in a small cohort of 73 patients, diarrhea was reported by 35.6% of the patients [22]. As for abdominal pain, its prevalence has been reported between 2.2 and 5.8% in different studies [5, 16]. The pooled prevalence estimates of diarrhea, nausea/vomiting, and abdominal pain were 7.7, 7.8, and 2.7% in a meta-analysis of 47 studies [23]. In the same meta-analysis, the corresponding pooled prevalence estimates of the aforementioned GI symptoms were 5.8, 5.2, and 2.7% in the studies from China, and 18.3, 14.9, and 5.3% in the studies from countries other than China [23]. The difference in sample size, the viral load of the patients in the GI system, and the different routes of transmission can be responsible for such variations.

Besides, the high prevalence of digestive symptoms, particularly nausea and/or vomiting in our study and some others is subject to significant confounding since nausea and vomiting are quite prevalent in viral illnesses and COVID-19 is no exception [24]. Further, the overall incidence rate of diarrhea in COVID-19 is reported to range from 2 to 50%. D’Amico et al. have justified the development of diarrhea in COVID-19 by elaborating on the role of ACE2 receptors [25]. Nonetheless, one reason for the variability of incidence rates may be different definitions of diarrhea, as similar to nausea/vomiting, diarrhea can occur with any viral illness. Viral diarrhea usually resolves within 48 h, while the best definition for diarrhea in the context of COVID-19 is at least 3 bowel movements lasting > 48 h [26]. Also, it is not clear whether diarrhea in COVID-19 results from the direct cytopathic effects of the virus, the cytokine storm altering the gut–brain axis, the altered gut flora caused by concomitant infections, the use of antimicrobials, or enteral feeding tubes, the use of antivirals for the treatment of COVID-19, or worsened underlying GI diseases such as inflammatory bowel disease, irritable bowel syndrome, and malabsorption syndromes [27–30].

We found that patients with both GI and respiratory symptoms were more prone to headache, fatigue, shivering, anosmia/ageusia, and fatigue compared to those with respiratory symptoms alone. This was in line with the findings of Jin et al. [31]. They also showed that patients with GI symptoms had increased CRP and neutrophils. Similarly, the percentage of neutrophils was significantly higher in the GIA group of our study compared to the RA and GIR groups; nevertheless, CRP levels were comparable in groups. Furthermore, liver damage has been reported in approximately one fifth of COVID-19 patients with GI symptoms along with elevated ALT, AST, and bilirubin levels, indicating liver dysfunction [31]. This was also consistent with our findings, in that although not significant, we found increased levels of liver function indicators in the GIA group compared to other groups of patients.

Prediction of progression into the severe form of the disease is of utmost importance in COVID-19 patients because it leads to proper preparation and resource allocation for patients at higher risk. It has been shown that GI symptoms increase the odds of severe COVID-19. In fact, Henry et al. demonstrated that abdominal pain was associated with approximately four-fold higher risk of severe COVID-19; nevertheless, this increased risk was marginal for nausea/vomiting and none for diarrhea. They argued that severe COVID-19 is accompanied by a high viral load leading to the significantly increased replication of the virus in the GI tract which may result in abdominal pain [32]. In the current study, all patients in the RA and WGIR groups had positive RT-PCR, while only 29.3% of the GIA and 53% of the GIR groups tested positive for SARS-CoV-2. This may show the higher load of virus in the GI tract in these patients since RT-PCR was performed on specimens obtained from the nasopharyngeal cavity; the increased distribution of SARS-CoV-2 in the GI tract can reduce the probability of its presence in the nasopharyngeal cavity. Nevertheless, this should be confirmed by RT-PCR on gastrointestinal specimens. With regard to the severity of COVID-19, we found that ICU admission, invasive ventilation, and oxygen therapy were more frequent in patients of the GIA group compared to those in the RA and GIR groups. In addition, aside from the high mortality in the WGIR group, the highest number of deaths occurred in the GIA group. Altogether, the findings of the current study are in harmony with the previous reports, all showing an association between the presence of GI symptoms and severity of COVID-19 [4, 5, 16, 33–35]. More importantly, almost all the previous studies found this association in patients with GI symptoms along with the respiratory symptoms of COVID-19; however, by categorizing the patients into four groups in the current study, we showed that when GI symptoms are the only manifestations of the disease, COVID-19 may be more severe, probably due to a higher viral load in the GI tract compared to when the virus is present in both the respiratory and GI systems. Again, these theories need to be confirmed by evaluation of the viral load in the respiratory and GI systems in both of these cases. Another reason for the increased severity of COVID-19 in patients with GI symptoms is that they may delay seeking medical attention due to the lack of respiratory symptoms. This has been confirmed by the findings of Pan et al. reporting a longer time to admission from onset of the disease in COVID-19 patients with digestive symptoms [15].

Another finding of the current study was that patients in the GIA group were significantly older than those in the RA and GRI groups, which is consistent with the findings of some studies [36], while other studies have shown that patients with GI symptoms are usually young and tend to have a benign course of the disease [37–39].

One strength of the current study was the comparison of CT findings between patients in the GIR and GIA groups which had not been performed in previous studies. As expected, aside from linear opacities and interstitial abnormalities, other CT findings were more frequent in the GIR group; yet, the differences were not statistically significant. This shows that although infrequently, COVID-19 patients who only present with GI symptoms can have lung involvement in the CT scan without any respiratory symptoms.

One limitation of the current study was that due to its retrospective design we were not able to determine the presenting symptoms of the patients. This is especially important in COVID-19 patients with both respiratory and GI symptoms; we were not aware of the sequence of symptom presentation as whether these patients presented with GI symptoms and later developed respiratory symptoms or the other way around. Another limitation was that approximately one fourth of the patients included in this study had negative RT-PCR results while they were marked as having COVID-19 with a high probability in their medical files based on clinical, laboratory, and CT findings collectively. However, the positivity of RT-PCR results largely depends on the timing of the test and these patients could have tested positive in a repeat RT-PCR. Besides, the RT-PCR results for these patients could have been false negative. Furthermore, the conventional RT-PCR is performed on nasopharyngeal specimens reflecting the colonization of the virus in the nasopharyngeal cavity. Since all negative RT-PCR results were in the GIA and GIR groups they might have tested positive if RT-PCR had been performed on stool specimens. Our inability to control the confounding factors due to its retrospective design was another limitation of this study.

## Conclusions

To sum up, the typical respiratory symptoms of COVID-19 are quite commonly accompanied by GI symptoms. With nausea and/or vomiting being the most prevalent, followed by diarrhea and abdominal pain as reported in the current study. Also, 18.1% of our patients only had GI symptoms. Noteworthy, a subgroup of COVID-19 patients may exclusively present with GI symptoms. This bears significance for both infection control and patient care. Clinicians and gastroenterologists should pay special attention to these patients in order to avoid underdiagnosis and delayed treatment or patient isolation.

## Data Availability

The datasets used and/or analyzed during the current study are available from the corresponding author on reasonable request.

## Abbreviations

ALT: Alanine aminotransferase
AST: Alanine aminotransferase
BUN: Blood urea nitrogen
COVID-19: Coronavirus disease 2019
Cr: Creatinine
CRP: C-reactive protein
DBP: Diastolic blood pressure
ESR: Erythrocyte sedimentation rate
G6PD: Glucose-6-phosphate dehydrogenase
GI: Gastrointestinal
GIA: Gastrointestinal symptoms alone
GIR: Gastrointestinal and respiratory symptoms
Hb: Hemoglobin
Hct: Hematocrit
HR: Heart rate
ICU: Intensive care unit
INR: International normalized ratio
K: Potassium
LDH: Lactate dehydrogenase
Mg: Magnesium
Na: Sodium
PG: Plasma glucose
PT: Prothrombin time
PTT: Partial thromboplastin time
RA: Respiratory symptoms alone
RR: Respiratory rate
RT-PCR: Reverse transcriptase polymerase chain reaction
SARS-CoV-2: Severe acute respiratory syndrome coronavirus 2
SBP: Systolic blood pressure
SD: Standard deviation
WBC: White blood cells
WGIR: Without gastrointestinal or respiratory symptoms
